# Rational thermostabilisation of four-helix bundle dimeric de novo proteins

**DOI:** 10.1038/s41598-021-86952-2

**Published:** 2021-04-06

**Authors:** Shin Irumagawa, Kaito Kobayashi, Yutaka Saito, Takeshi Miyata, Mitsuo Umetsu, Tomoshi Kameda, Ryoichi Arai

**Affiliations:** 1 Department of Science and Technology, Graduate School of Medicine, Science and Technology, Shinshu University, Ueda, Nagano 386-8567 Japan; 2 Department of Biomolecular Innovation, Institute for Biomedical Sciences, Interdisciplinary Cluster for Cutting Edge Research, Shinshu University, Matsumoto, Nagano 390-8621 Japan; 3 Department of Applied Biology, Faculty of Textile Science and Technology, Shinshu University, Ueda, Nagano 386-8567 Japan; 4 Artificial Intelligence Research Center, National Institute of Advanced Industrial Science and Technology (AIST), Tokyo, 135-0064 Japan; 5 AIST-Waseda University Computational Bio Big-Data Open Innovation Laboratory (CBBD-OIL), Tokyo, 169-8555 Japan; 6 Graduate School of Frontier Sciences, The University of Tokyo, Kashiwa, Chiba 277-8561 Japan; 7 Department of Biochemistry and Biotechnology, Faculty of Agriculture, Kagoshima University, Kagoshima, 890-0065 Japan; 8 Department of Biomolecular Engineering, Graduate School of Engineering, Tohoku University, Sendai, 980-8579 Japan

**Keywords:** Biochemistry, Biochemistry, Biological physics

## Abstract

The stability of proteins is an important factor for industrial and medical applications. Improving protein stability is one of the main subjects in protein engineering. In a previous study, we improved the stability of a four-helix bundle dimeric de novo protein (WA20) by five mutations. The stabilised mutant (H26L/G28S/N34L/V71L/E78L, SUWA) showed an extremely high denaturation midpoint temperature (*T*_m_). Although SUWA is a remarkably hyperstable protein, in protein design and engineering, it is an attractive challenge to rationally explore more stable mutants. In this study, we predicted stabilising mutations of WA20 by in silico saturation mutagenesis and molecular dynamics simulation, and experimentally confirmed three stabilising mutations of WA20 (N22A, N22E, and H86K). The stability of a double mutant (N22A/H86K, rationally optimised WA20, ROWA) was greatly improved compared with WA20 (Δ*T*_m_ = 10.6 °C). The model structures suggested that N22A enhances the stability of the α-helices and N22E and H86K contribute to salt-bridge formation for protein stabilisation. These mutations were also added to SUWA and improved its *T*_m_. Remarkably, the most stable mutant of SUWA (N22E/H86K, rationally optimised SUWA, ROSA) showed the highest *T*_m_ (129.0 °C). These new thermostable mutants will be useful as a component of protein nanobuilding blocks to construct supramolecular protein complexes.

## Introduction

Improving the stability of proteins is one of main subjects in protein engineering. Development of methods to stabilise proteins will contribute to medical and industrial applications. For many years, stabilisation of various proteins has been studied in the fields of protein engineering^1–6^. Many studies on protein stabilisation by mutagenesis have been reported, but investigating sufficient mutations of amino-acid residues requires enormous effort and cost for experiments. Several computational methods have been used to rationally predict stabilising mutations^7,8^. Because proteins are dynamic molecules in solution and molecular dynamics (MD) simulation provides information about the dynamic behaviour of molecules in variable environments in silico, MD simulation is useful for designing stabilised proteins^9^.

Protein design is also a main and hot topic in protein engineering^10–13^. As a semirational approach for protein design, the binary code strategy was developed to construct libraries of novel polypeptides (de novo proteins) that would fold into predetermined structures^14^. Using secondary structure motifs with binary patterns of polar and nonpolar residues, de novo proteins with α-helices or β-sheets have been successfully created without reference to natural protein sequences. WA20 is one of the de novo proteins obtained from a library of binary patterned four-helix bundles^15^. Previously the crystal structure of WA20 was solved, revealing an intermolecularly folded dimeric four-helix bundle (PDB ID: 3VJF)^16^ with a bisecting U topology^17^. Moreover, utilising the characteristic dimeric structure of WA20 as a component, protein nanobuilding blocks (PN-Blocks)^18,19^ were developed to construct self-assembled supramolecular nanostructures: WA20-foldon, constructed by fusing dimeric WA20 to a trimeric foldon domain of T4 phage fibritin formed several types of self-assembled nanoarchitectures, including a barrel-like hexamer and a tetrahedrally shaped dodecamer^18^. In addition, extender protein nanobuilding blocks (ePN-Blocks) was constructed by tandemly joining two copies of WA20 with various linkers^19^. The ePN-Blocks self-assembled into cyclised and extended chain-type nanostructures.

With the long-term goal of producing nanostructures with extremely high stabilities for applications in nanotechnology, we were motivated to stabilise the designed protein WA20, a main component of PN-Blocks. Recently, we succeeded in dramatically improving the stability of WA20 by introducing five amino acid substitutions (H26L, G28S, N34L, V71L, and E78L)^20^ to enhance the hydrophobic core and α-helix stability based on the WA20 structure. This mutant, which is called super WA20 (SUWA), showed an extremely high denaturation midpoint temperature (*T*_m_) above the boiling point of water.

Although SUWA is a remarkably hyperstable protein, in protein engineering, it is an attractive challenge to rationally explore more stable mutants than the de novo designed proteins WA20 and SUWA. In this study, we performed rational design of new mutations to stabilise WA20 and SUWA by in silico mutagenesis and MD simulation. Three mutations (N22A, N22E, and H86K) were found to improve *T*_m_ of WA20. A double mutant of WA20 (N22A/H86K, which is called rationally optimised WA20, ROWA) greatly improved *T*_m_ by 10.6 °C. Moreover, these mutations improved the thermostability of SUWA. In particular, a double mutant of SUWA (N22E/H86K, which is called rationally optimised SUWA, ROSA) showed the highest *T*_m_ of 129.0 °C. In addition, the ROWA and ROSA oligomers were characterised by size exclusion chromatography–multi-angle light scattering (SEC–MALS) and small-angle X-ray scattering (SAXS).

## Results

### Prediction of mutations to stabilise WA20

**Figure 1:**
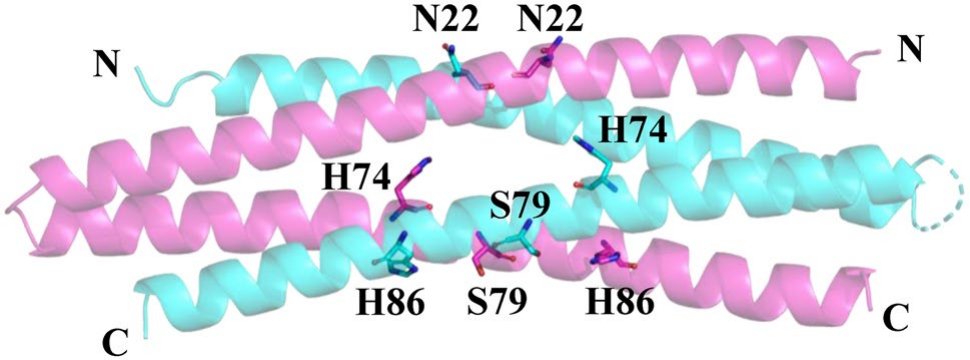
Target residues for mutations to stabilise the de novo protein WA20 in the present study. The target amino acid residues are shown as sticks. Chains A and B of the crystal structure of WA20 (PDB: 3VJF)^16^ are shown in magenta and cyan, respectively. The images were created using open- source PyMOL, version 2.4 (https://github.com/schrodinger/pymol-open-source).

For soluble and stable proteins, generally, most of the amino acid residues on the surface of the proteins are hydrophilic and most of the residues inside the proteins are hydrophobic. However, in some cases, there are some unusual residues, which are hydrophilic residues buried inside the protein, that can be potentially optimised. Therefore, to select target residues for mutations, we searched for hydrophilic residues buried in the WA20 protein structure based on the accessible surface area (ASA) per residue (Supplementary Table S1). Five hydrophilic residues buried inside the structure of WA20 (H26, H74, E78, S79, and H86) were found based on small ASA ratios (ASA ratio ≤ 0.11 in both the A and B chains). In addition, we chose two target residues (N22 and N34) on the interface of the α-helices to potentially enhance the helix–helix interactions (Supplementary Fig. S1). In a previous study of SUWA^20^, we tested some mutations (H26L/E78L and N34L) at these target residue sites. The double mutation H26L/E78L significantly improved *T*_m_ of WA20 by 26 °C and the single mutation N34L improved *T*_m_ by 10°C^20^. Therefore, in the present study, we focused on the target residues (N22, H74, S79, and H86, Fig. 1) that we did not test previously.

Saturation mutagenesis is an exhaustive search for all possible mutations of 19 amino acids at a target site, but enormous effort and cost are required for experiments. Hence, we attempted to predict stabilising mutations using MD simulation. First, we performed high-temperature MD simulations of WA20 and the mutants constructed in a previous study (G28S, N34L, V71L, H26L/E78L, H26L/G28S/V71L/E78L, and SUWA)^20^. The root mean square deviations (RMSDs) between the initial structures and the structures after high-temperature MD simulations were calculated to evaluate the degree of protein unfolding. The RMSD values of WA20 and the mutants showed a strong negative correlation with *T*_m_ of the mutants (*r* =  − 0.966, *p* = 5.461 × 10^−5^, Supplementary Fig. S2), suggesting that RMSD is a useful indicator to evaluate the stability of mutants. Therefore, we performed high-temperature MD simulations of all of the possible single mutants and the original WA20 (i.e., in silico saturation mutagenesis) at the four target residue sites (N22, H74, S79, and H86). Based on the MD simulation results, we selected eight mutations of WA20 with small RMSD values (N22A, N22E, N22K, N22L, H86K, H86S, H74V, and S79F, Supplementary Fig. S3) for subsequent experiments. At the N22 site, the N22G mutation showed the smallest RMSD value, but we did not adopt N22G because the RMSD values of N22G showed relatively large variability and glycine is generally unfavourable in an α-helix of proteins^21^. Because the H74 and S79 sites are close, we constructed the double mutant of H74V/S79F with one oligo-DNA primer for the site-directed mutations.

### Development of stabilised mutants of WA20

**Figure 2:**
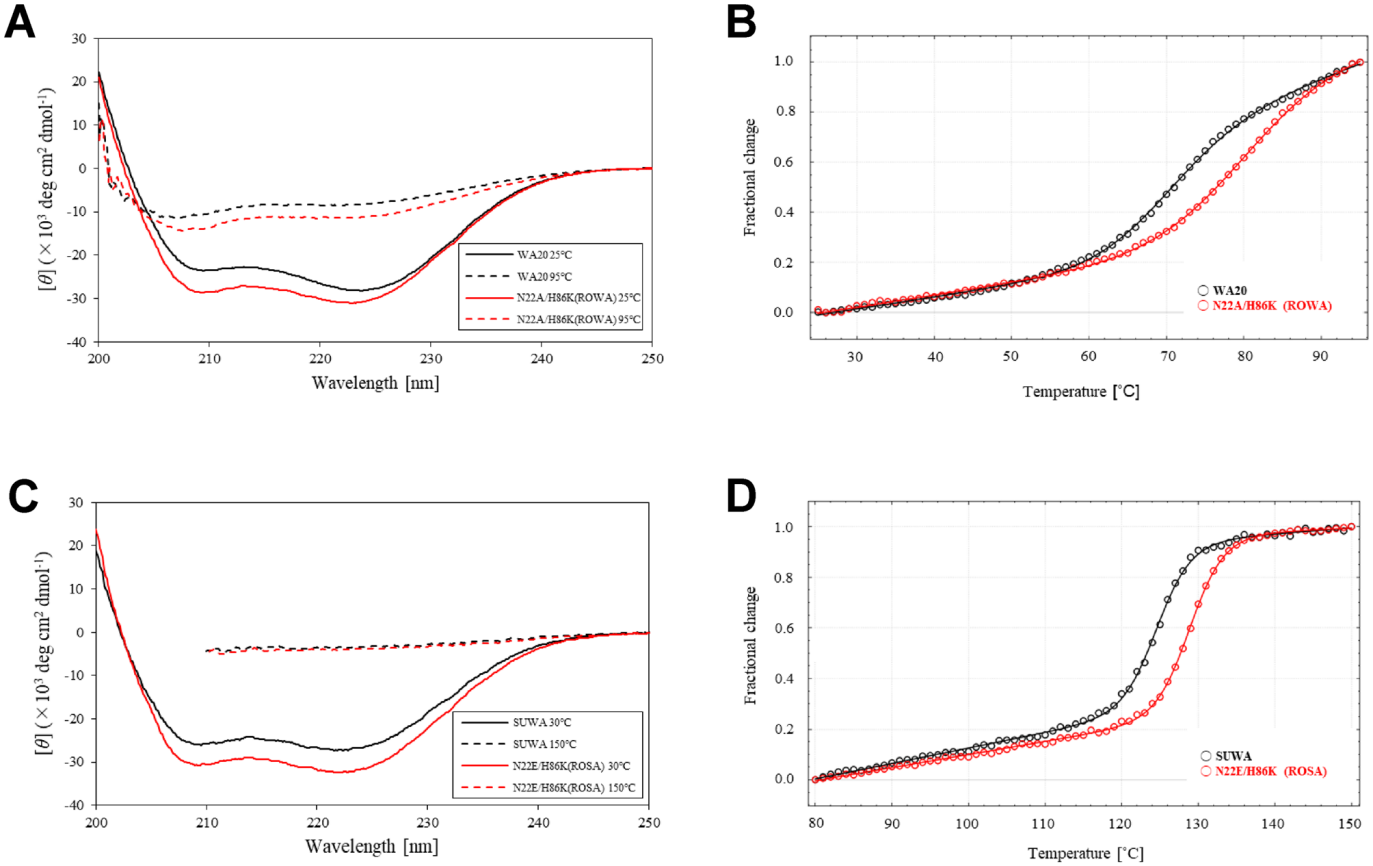
CD spectra and thermal denaturation curves. (**A**) CD spectra of WA20 and the N22A/H86K mutant of WA20 (ROWA) at 25 and 95 °C. (**B**) Thermal denaturation curves of WA20 and ROWA. The *T*_m_ values of the WA20 and ROWA proteins are 69.8 ± 0.4 and 80.4 ± 3.5 °C, respectively. (**C**) CD spectra of SUWA and the N22E/H86K mutant of SUWA (ROSA) at 30 and 150 °C. (**D**) Thermal denaturation curves of SUWA and ROSA. The *T*_m_ values of SUWA and ROSA are 124.7 ± 0.1 and 129.0 ± 0.1 °C, respectively. In the denaturation curves, the *Y-*axis represents the normalised value of [*θ*]_222 nm_. The data of the denaturation curves were fitted to a two-state model with Δ*C*_p_ fixed to zero and the graphs were created using CDpal^33^, version 2.18 (https://github.com/PINT-NMR/CDpal/).

WA20 and the mutant proteins were expressed in *Escherichia coli* and purified by immobilised metal ion affinity chromatography (IMAC) (Supplementary Fig. S4). The circular dichroism (CD) spectra at 25 °C showed that all of the mutants formed α-helical structures, as well as the parental WA20 protein (Fig. 2A and Supplementary Fig. S5).

Thermal denaturation experiments (Table 1 and Supplementary Fig. S6) revealed that the N22A, N22E, and H86K mutants had higher midpoint temperatures than WA20 (Δ*T*_m_ of 2.9 °C for N22A, 1.0 °C for N22E, and 3.5 °C for H86K). In contrast, the N22K and N22L mutations considerably reduced the stability (Table 1).

**Table 1:** Denaturation midpoint temperatures (*T*_m_) of the WA20 mutants.

Sample	*T*_m_ (°C)	*ΔT*_m_ (°C)
WA20	69.8 ± 0.4	–
N22A	72.7 ± 0.6	2.9
N22E	70.8 ± 0.5	1.0
N22K	60.9 ± 1.0	− 8.9
N22L	65.0 ± 0.9	− 4.8
H86K	73.3 ± 0.7	3.5
H86S	68.6 ± 0.8	− 1.2
H74V/S79F	69.9 ± 1.2	0.1
N22A/H86K (ROWA)	80.4 ± 3.5	10.6
N22E/H86K	71.7 ± 1.1	1.9

To test combination of these stabilising mutations, we constructed two double mutants of WA20: N22A/H86K and N22E/H86K. The N22A/H86K mutant (ROWA), remarkably improved the stability (Δ*T*_m_ = 10.6 °C) compared with the parental WA20 protein (Fig. 2B and Table 1).

### Development of stabilised mutants of SUWA

We also added the three stabilising mutations (N22A, N22E, and H86K) to SUWA, which is the hyperstable mutant (H26L/G28S/N34L/V71L/E78L) of WA20 constructed in a previous study^20^. Three single mutants (N22A, N22E, and H86K) and two double mutants (N22A/H86K and N22E/H86K) of SUWA were constructed, expressed in *E. coli*, and purified by IMAC (Supplementary Fig. S7). For the SUWA_N22E and SUWA_N22E/H86K mutants, the yields of the purified proteins improved compared with SUWA (amount of purified protein per litre of culture of ~ 22 mg for SUWA_N22E, ~ 19 mg for SUWA_N22E/H86K, and ~ 6 mg for SUWA). The CD spectra at 30 °C showed that all of the mutants of SUWA formed α-helical structures, as well as the SUWA protein (Fig. 2C and Supplementary Fig. S8). Thermal denaturation experiments revealed that all of the mutants of SUWA had higher midpoint temperatures than SUWA (Table 2, Fig. 2D, and Supplementary Fig. S6D). Compared with SUWA, the single mutations N22A, N22E, and H86K improved *T*_m_ by 3.8, 1.1, and 0.9 °C, and the double mutations N22A/H86K and N22E/H86K improved *T*_m_ by 3.8 and 4.3 °C, respectively. We call the mutant with the highest *T*_m_ (SUWA_N22E/H86K, *T*_m_ = 129.0 °C) ROSA.

**Table 2:** Denaturation midpoint temperatures (*T*_m_) of the SUWA mutants.

Sample	*T*_m_ (°C)	Δ*T*_m_ (°C)
SUWA	124.7 ± 0.1	–
SUWA_N22A	128.5 ± 0.1	3.8
SUWA_N22E	125.8 ± 0.1	1.1
SUWA_H86K	125.6 ± 0.3	0.9
SUWA_N22A/H86K	128.5 ± 0.5	3.8
SUWA_N22E/H86K (ROSA)	129.0 ± 0.1	4.3

### Characterisation of ROWA and ROSA oligomers

**Figure 3:**
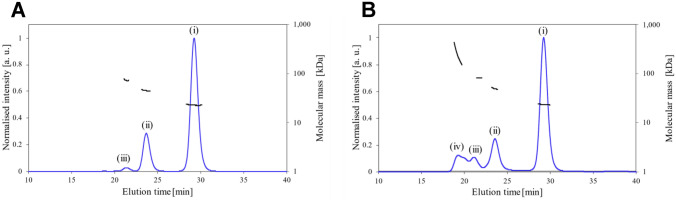
SEC–MALS analysis. SEC–MALS profiles of (**A**) ROWA and (**B**) ROSA. The blue lines and the black lines represent the normalised intensity of UV absorbance (*A*_280nm_) and the molecular mass of the protein oligomers for each peak, respectively.

To evaluate the oligomeric states of the ROWA and ROSA proteins in solution, SEC–MALS experiments were performed (Fig. 3). The molecular mass for each peak of the ROWA and ROSA proteins was determined (Table 3). The oligomeric number of each peak was calculated using the theoretical molecular mass values of monomers of ROWA (12.5 kDa) and ROSA (12.6 kDa). The ROWA protein mainly formed a dimer (peak (i), 77%), and it also formed a tetramer (peak (ii), 21%) and a hexamer (peak (iii), 2%). The ROSA protein mainly formed a dimer (peak (i), 64%), and it also formed a tetramer (peak (ii), 18%), a hexamer (peak (iii), 7%), and higher oligomers (peak (iv), 11%). Although the parental WA20 and SUWA proteins only formed dimers^16,20^, the ROWA and ROSA proteins formed not only dimers, but also some larger oligomers (tetramers, hexamers, and higher oligomers), suggesting that the introduced mutations increase the interactions forming larger oligomers.

**Table 3:** Summary of the SEC–MALS results of ROWA and ROSA.

Sample (peak)	*M*_w_ (kDa)	Oligomeric number (mer)	Mass fraction (%)
ROWA (i)	22.8	2	77
ROWA (ii)	45.3	4	21
ROWA (iii)	72.8	6	2
ROSA (i)	23.4	2	64
ROSA (ii)	49.2	4	18
ROSA (iii)	81.2	6	7
ROSA (iv)	238	19	11

In addition, the SEC profiles of the isolated dimer peak fractions of ROWA and ROSA did not change after one week (Supplementary Fig. S9), suggesting that the oligomeric states are stable and do not exchange to the other oligomeric states on a timescale of a week at room temperature.

### SAXS analysis

**Figure 4:**
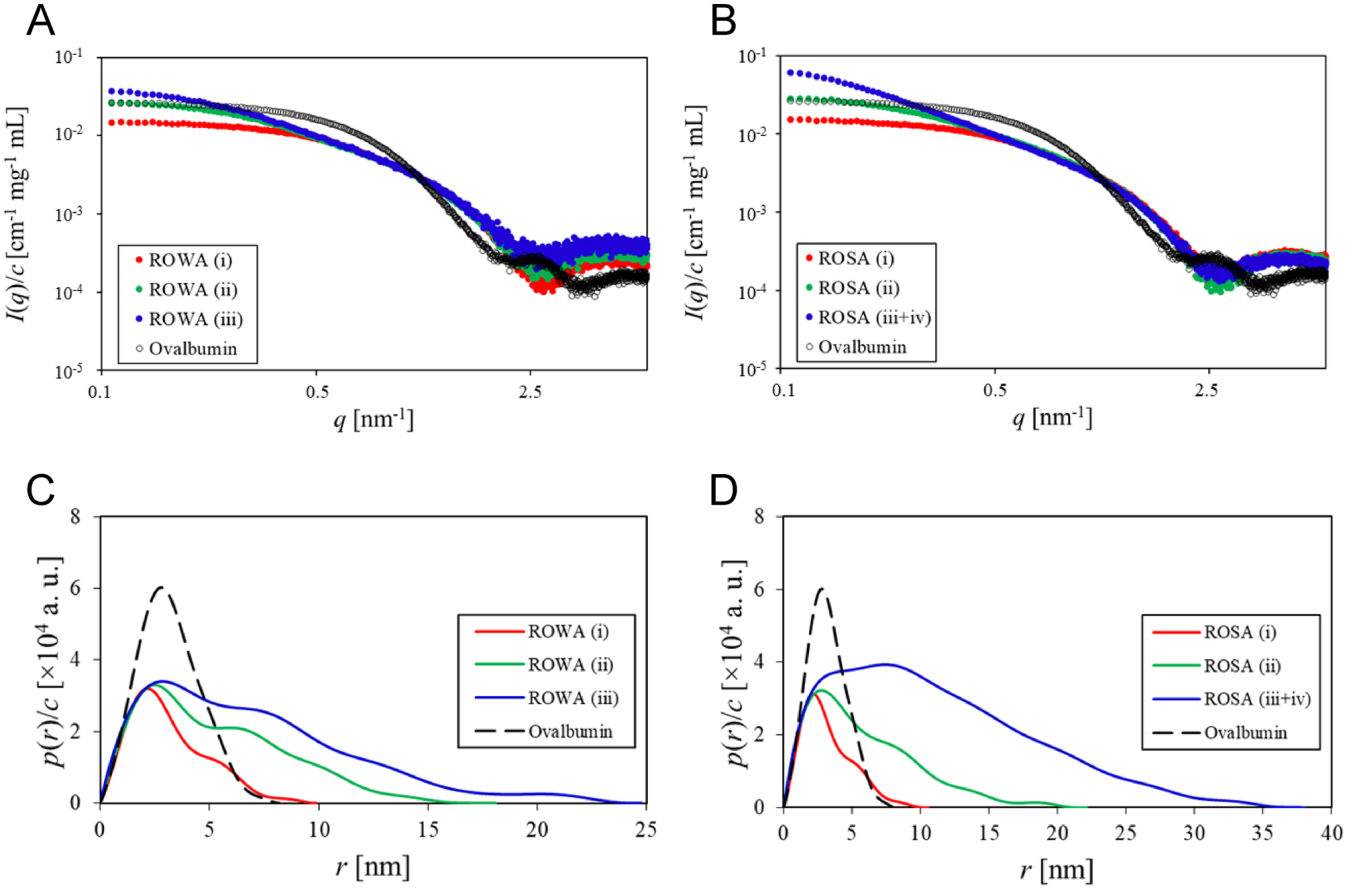
SAXS analysis. Concentration-normalised absolute scattering intensities of the (**A**) ROWA and (**B**) ROSA samples. Concentration-normalised pair-distance distribution functions of the (**C**) ROWA and (**D**) ROSA samples. The samples of the eluted peaks were fractionated by SEC purification as shown in Fig. 3. Ovalbumin was used as a reference standard of the molecular mass.

To further analyse the ROWA and ROSA oligomers, we performed SAXS experiments (Fig. 4A,B) of the samples fractionated by SEC purification. The weight-average molecular mass (*M*_w_) values of the ROWA and ROSA samples (except for ROSA (iii + iv)) (Table 4) calculated from Guinier plots (Supplementary Fig. S10) were consistent with those from the SEC–MALS experiments (Table 3).

**Table 4:** Summary of the SAXS results of ROWA and ROSA.

Sample	*I*(*q* → 0)/*c* (cm^−1^ mg^−1^ mL)	*D*_max_ (nm)	*R*_g_ (nm)	*M*_w_ (kDa)
ROWA (i)	0.01501	9.90	2.45	25.0
ROWA (ii)	0.02804	18.1	4.09	46.8
ROWA (iii)	0.04034	24.8	5.25	67.3
ROSA (i)	0.01497	10.6	2.50	25.0
ROSA (ii)	0.03022	22.1	4.42	50.4
Ovalbumin	0.02657	8.19	2.45	44.3^a^

^a^Ovalbumin was used as a reference standard of the molecular mass.

To extract intuitive real-space information from the SAXS data, we obtained the pair-distance distribution functions (*p*(*r*)) reflected by the shapes of the ROWA and ROSA oligomers by the indirect Fourier transformation (IFT) technique (Fig. 4C,D). The shapes of *p*(*r*) for all of the samples of ROWA and ROSA were characterised by an extended tail in the high-*r* region, suggesting formation of rod-like elongated shapes similar to WA20 and SUWA. The *D*_max_ values of ROWA (i) (dimer), ROWA (ii) (tetramer), ROWA (iii) (hexamer), ROSA (i) (dimer), ROSA (ii) (tetramer), and ROSA (iii + iv) (hexamer and higher oligomers) indicated that the larger oligomers formed longer shapes (Fig. 4C,D and Table 4).

**Figure 5:**
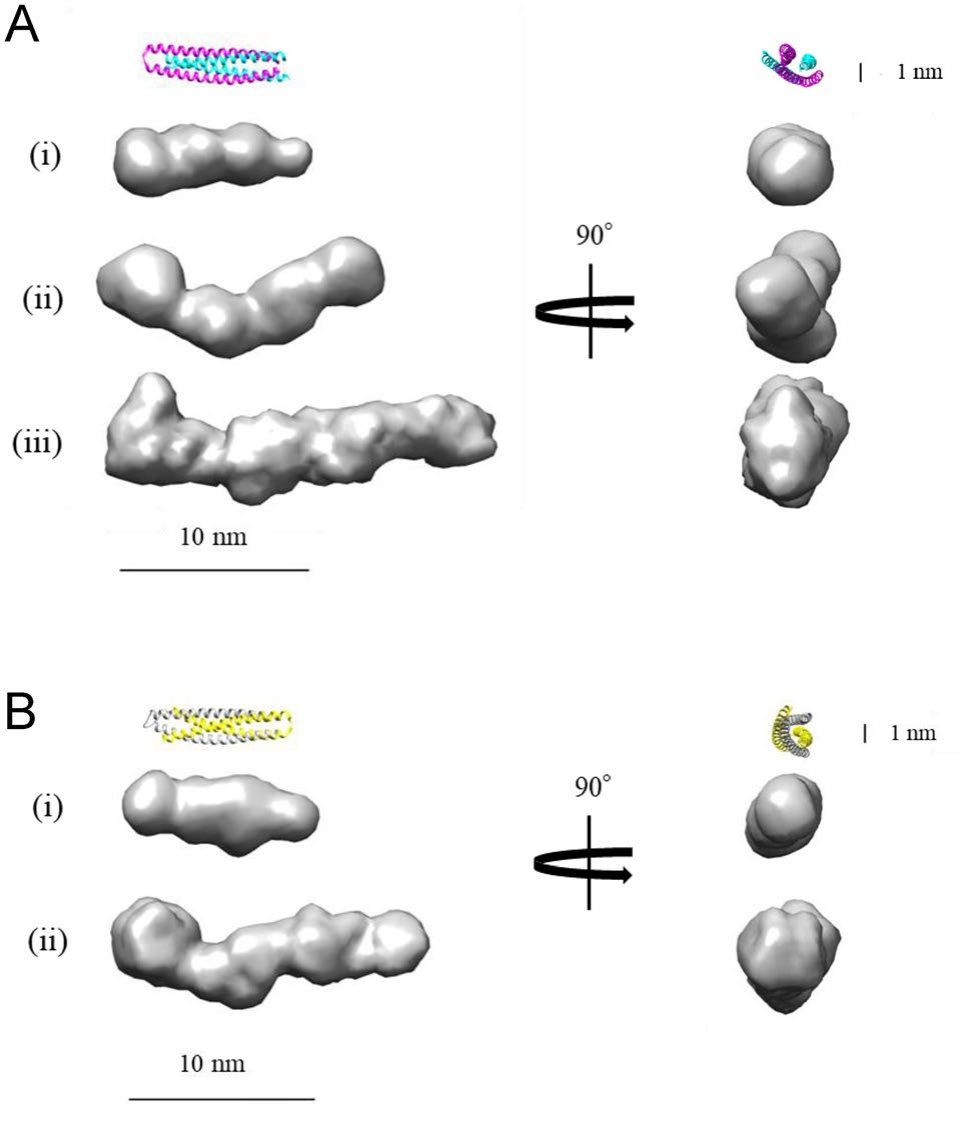
Dummy atom model shapes of the ROWA and ROSA oligomers. (**A**) Dummy atom models of the ROWA (i) dimer, (ii) tetramer, and (iii) hexamer. (**B**) Dummy atom models of the ROSA (i) dimer and (ii) tetramer. The models were constructed based on the SAXS data using the ab initio modelling programs DAMMIF^40^, DAMAVER^41^, and DAMMIN^42^ without symmetry constraints. Ribbon representations of the crystal structures of WA20 (PDB ID: 3VJF)^16^ and SUWA (PDB ID: 6KOS)^20^ are shown as references. These images were created using UCSF Chimera^43^, version 1.12 (https://www.cgl.ucsf.edu/chimera/). The SAXS data and dummy atom models have been deposited into SASBDB^44,45^.

Low-resolution dummy atom models were reconstructed from the SAXS data (Fig. 5 and Supplementary Fig. S11). The ab initio dummy atom models of the ROWA and ROSA dimers (Fig. 5A(i),B(i)) were almost the same shapes as structures of the WA20 and SUWA dimers. Interestingly, the dummy atom models of the ROWA and ROSA tetramers (Fig. 5A(ii),B(ii)) and the ROWA hexamer (Fig. 5A(iii)) were more elongated than those of the dimers.

## Discussion

Thermal denaturation is one of the main problems that limit industrial and medical use of proteins. However, experimental searches for stabilising mutations by saturation mutagenesis require enormous effort and cost because there are potentially 19 candidate amino acids at each target residue site. In this study, we attempted to discover rationally stabilising mutations of the de novo protein WA20 by MD simulation. At the four target residue sites (N22, H74, S79, and H86), we selected seven mutations of WA20 (N22A, N22E, N22K, N22L, H86K, H86S, and H74V/S79F) based on in silico saturation mutagenesis and high-temperature MD simulations. Three mutations improving *T*_m_ of WA20 (N22A, N22E, and H86K, Table 1) were found. We then examined combinations of these mutations. While both double mutations (N22A/H86K and N22E/H86K) stabilised WA20, the contributions of these mutations to thermostabilisation may be different (Table 1). Because Δ*T*_m_ of N22A/H86K (10.6 °C) is greater than the sum of the Δ*T*_m_ values of N22A (2.9 °C) and H86K (3.5 °C), combination of these mutations synergistically contributes to the protein stability. In contrast, Δ*T*_m_ of N22E/H86K (1.9 °C) is less than Δ*T*_m_ of H86K (3.5 °C), indicating that the combination of N22E and H86K may have a negative effect for the stability of WA20.

We also added the three mutations (N22A, N22E, and H86K) to SUWA, which is the hyperstable mutant of WA20 with five mutations (H26L, G28S, N34L, V71L, and E78L) developed in a previous study^20^. Three single mutations (N22A, N22E, and H86K) and two double mutations (N22A/H86K and N22E/H86K) of SUWA improved *T*_m_ (Table 2). In particular, the combination of N22E and H86K mutations (ROSA) greatly improved the thermostability. As in the case of ROSA, Δ*T*_m_ of N22E/H86K (4.3 °C) is greater than the sum of the Δ*T*_m_ values of N22E (1.1 °C) and H86K (0.9 °C), suggesting that the combination of these mutations synergistically contributes to the protein stability. These results suggest that MD simulation is useful for finding stabilising mutations.

Structural models of the mutants (N22A, N22E, and H86K) of WA20 and SUWA suggest possible mechanisms for stabilisation of the structures (Supplementary Fig. S12). In the structures of the WA20 and SUWA dimers, two N22 residues are located near the R25 residues on the interface of the helices in both chains A and B (Supplementary Fig. S12A). In many natural proteins, alanine is the amino acid that has a preference to make an α-helix^21^. The N22A mutation can promote formation of α-helices and improve the structural stability. In contrast, the N22E mutation can electrostatically stabilise the protein structure by intra- and inter-chain interactions with R25 (Supplementary Fig. S12A).

Conversely, the N22K and N22L mutations destabilise the WA20 protein. Two lysine residues (N22K) and two arginine residues (R25) electrostatically repel one another (Supplementary Fig. S13). The leucine residues (N22L) may destabilise the structure probably because of exposure of large hydrophobic side chains on the interface of the helices to the solution (Supplementary Fig. S13).

In both the WA20 and SUWA structures, H86 interacts with D72 (Supplementary Fig. S12B). In neutral pH conditions, lysine has more positive charge than histidine, and thus the lysine residues (H86K) form stronger ionic bonds with the aspartic acid residues (D72), contributing to the thermostability (Supplementary Fig. S12B).

Several stabilised mutants of WA20 and SUWA, such as ROWA and ROSA developed in this study, have advantages for constructing supramolecular complexes of protein nanobuilding blocks (PN-Blocks)^18,19^. In particular, the ROSA protein achieved extremely high thermostability (*T*_m_ = 129.0 °C) and the protein expression level of ROSA in *E. coli* improved compared with SUWA. Therefore, ROSA is expected to improve the thermal stability and productivity of PN-Blocks, contributing to protein engineering and synthetic biology.

## Methods

### Selection of the target residue sites for mutations

Candidates for the target residues for mutations of WA20 were selected based on the ASA (Table S1) in the crystal structure of WA20 (PDB ID: 3VJF)^16^ calculated by the program AREAIMOL^22,23^ in the CCP4 suite^24^. The hydrophilic residues (H26, H74, E78, S79, and H86) buried inside were selected (ASA ratio to calculated GXG value ≤ 0.11). In addition, target residues on the interface of α-helices (N22 and N34) were selected by manual inspection of the crystal structure of WA20 to potentially enhance helix–helix interactions. Because some mutations (H26L/E78L and N34L) at the candidate residue sites had already been tested in a previous study of SUWA^20^, in the present study, we investigated the target residues (N22, H74, S79, and H86) that had not been previously tested.

### MD simulation

The mutant structures were generated from the crystal structure of WA20 (PDB ID: 3VJF)^16^ or SUWA (PDB ID: 6KOS)^20^ using the Scwrl4 program^25^. The MD simulations were performed with the GROMACS 2016 molecular simulation package^26^. The proteins were protonated and dissolved in a dodecahedral box and placed at least 2.0 nm from the box edges. Periodic boundary conditions were applied in all directions. The box was filled with water molecules. Sodium and chloride ions were added to each box to neutralise the total charge. The AMBER ff14SB force field^27^ was used to represent the proteins and the TIP3P model^28^ was used for water. After energy minimisation, constant-pressure and constant-temperature (NPT) MD simulations were performed at 1 bar and 300 K for 0.1 ns, and then the production runs were performed at 600 K for 10 ns with ten random seeds for each mutant in the NVT ensemble (constant temperature and volume). The Berendsen method was used to maintain the pressure during the NPT simulation^29^. Langevin dynamics was used to control the temperature with water viscosity set to 2 ps^−1^. The covalent bonds of the hydrogen atoms in the proteins were constrained using the LINCS method^30^, and the integration time step was 2.0 fs. During the production run, the coordinates were saved every 100 ps. The RMSD between the structures before and after the production runs was calculated by the GROMACS tool.

### Construction of protein expression plasmids

The protein expression plasmids of the mutants of WA20 and SUWA were prepared by site-directed mutagenesis of the plasmid pET3-WA20^16^ or pET3-SUWA^20^ using the transfer-PCR method^31^ with the oligo-DNA primers (Table S2) and KOD-Plus-Neo DNA polymerase (Toyobo, Osaka, Japan). The amino acid sequences of the WA20, ROWA, SUWA, and ROSA proteins are shown in Supplementary Fig. S14.

### Protein expression and purification

The WA20, SUWA, and mutant proteins were expressed in *E. coli* BL21 Star (DE3) (Invitrogen, Carlsbad, CA, USA) harbouring an expression plasmid in 1 L of LB broth, Lennox (Nacalai Tesque, Kyoto, Japan) containing 100 µg/mL ampicillin sodium salt at 37 °C for 16 h. All the proteins were expressed in *E. coli* without isopropyl β-d-thiogalactopyranoside induction. The proteins were extracted from the harvested cells by freezing–thawing and sonication with a VC 505 ultrasonic processor (Sonics and Materials, Newtown, CT, USA) in 50 mM sodium phosphate buffer (pH 7.0) containing 300 mM NaCl and 10% glycerol. The proteins were purified by immobilised metal affinity chromatography (IMAC) with TALON metal affinity resin (Takara Bio, Kusatsu, Shiga, Japan). Because many histidine residues are exposed on the surface of the WA20 and SUWA structures^16,20^, WA20, SUWA, and their mutants without His-tag can bind to the IMAC resin. The resin was washed with 50 mM sodium phosphate buffer (pH 7.0) containing 300 mM NaCl and 10% glycerol, and the proteins were eluted with 50 mM sodium phosphate buffer (pH 7.0) containing 300 mM NaCl, 10% glycerol, and 200 mM imidazole. The protein concentration was determined by absorbance at 280 nm using a NanoDrop Lite spectrophotometer (Thermo Fisher Scientific, Waltham, MA, USA). The molar extinction coefficient of each protein was calculated according to the amino acid sequence (Trp: 5559, Tyr: 1197, and Phe: 0.7)^32^.

### CD spectroscopy

For the thermal denaturation experiments, we used a J-1500 spectropolarimeter (JASCO, Hachioji, Tokyo, Japan) specially equipped with a programmable temperature controller and a pressure-proof cell compartment that prevented the aqueous solution from boiling and evaporating at high temperatures. Thermal denaturation was monitored at the ellipticity of a typical peak of the α-helices of proteins (*θ*_222nm_) using the cell compartment pressured by nitrogen gas (+ 0.5 MPa). Each protein (∼0.3 mg/mL) was dissolved in 20 mM sodium phosphate buffer (pH 7.5) containing 150 mM NaCl and 10% glycerol. A cell with 0.1 cm path length was used. The temperature was increased at a rate of 2.0 °C/min. The thermal denaturation curves were analysed to calculate the denaturation midpoint temperatures (*T*_m_) using CDpal^33^, version 2.18. The data were fitted to a two-state model with Δ*C*_p_ fixed to zero. The errors were estimated by the CDpal program using the robust jackknife method^33^.

### SEC–MALS

The SEC–MALS experiments were performed using an Alliance e2695 HPLC system (Waters, Milford, MA, USA) equipped with a Superdex 75 Increase 10/300 GL column (Cytiva, Little Chalfont, Buckinghamshire, UK), which was connected in line with a DAWN HELEOS II multi-angle static light scattering detector (Wyatt Technology, Santa Barbara, CA). The data were collected at 20 °C with 20 mM HEPES buffer (pH 7.5) containing 150 mM NaCl and 5% glycerol and analysed using ASTRA 6 software (Wyatt Technology)^34^. The d*n*/d*c* value of 0.185 mL/g was generally used for the proteins with extinction coefficients of 0.542 mL mg^−1^ cm^−1^ for ROWA and 0.539 mL mg^−1^ cm^−1^ for ROSA calculated according to the amino acid sequences.

### SAXS

For the SAXS experiments, the ROWA and ROSA samples after IMAC purification were further purified by SEC (20 mM HEPES buffer (pH 7.5) containing 150 mM NaCl and 5% glycerol) with a Superdex 75 Increase 10/300 GL column (Cytiva). SAXS measurements were performed for samples (~ 1–4 mg/mL) of ROWA, ROSA, and chicken ovalbumin (A7641; Sigma-Aldrich, St. Louis, MO, USA) dissolved in the HEPES buffer at 20 °C using synchrotron radiation (λ = 1.3 Å) at the Photon Factory BL-10C beamline^35^ (KEK, Tsukuba, Japan) with a PILATUS3 2 M detector (Dectris, Baden, Switzerland) at a sample-detector distance of 1.08 m.

The two-dimensional scattering images were integrated into one-dimensional scattering intensities *I*(*q*) as a function of the magnitude of the scattering vector *q* = (4π/λ)sin(*θ*/2) using SAngler^36^, where *θ* is the total scattering angle.

The IFT technique was used to calculate the pair-distance distribution function *p*(*r*) using GNOM^37^ in the ATSAS program suite^38^. The forward scattering intensity, *I*(*q* → 0), and radius of gyration, *R*_g_, were estimated by Guinier approximation^39^ using AUTORG in ATSAS^38^ with SAngler^36^. Assuming that the proteins have practically the same scattering length density and specific volume and that the structure factor is almost unity (*S*(*q*) ≈ 1) for the dilute samples, the forward scattering intensity normalised by the protein concentration (mg/mL), *I*(*q* → 0)/*c*, is proportional to the weight average molecular mass (*M*_w_). Ovalbumin (*M*_w_ = 44.3 kDa) was used as a reference standard of the molecular mass.

The low-resolution dummy atom models were constructed from the SAXS data using the ab initio shape modelling programs in the ATSAS program suite^38^ for small-angle scattering data analysis from biological macromolecules. The calculations of rapid ab initio shape determination were performed ten times by DAMMIF^40^ without a symmetry constraint, and the generated models were aligned and averaged by DAMAVER^41^. The averaged model was modified with the fixed core by DAMSTART and further refinement of the model was performed by DAMMIN^42^. The images of the dummy atom models were prepared using UCSF Chimera^43^, version 1.12. The SAXS data and dummy atom models of ROWA and ROSA have been deposited into Small Angle Scattering Biological Data Bank (SASBDB)^44,45^ (accession codes: SASDKM8 for ROWA dimer, SASDKN8 for ROWA tetramer, SASDKP8 for ROWA hexamer, SASDKQ8 for ROSA dimer, and SASDKR8 for ROSA tetramer).

### Modelling of the mutant structures

The model structures of the mutants were constructed based on the crystal structure of WA20 (PDB ID: 3VJF)^16^ or SUWA (PDB ID: 6KOS)^20^ using the Scwrl4 program^25^. The model structures were optimised by MD simulation at 300 K for 1 ns. The structure images were created using open-source PyMOL, version 2.4 (Schrödinger, New York, NY, USA).

## Supplementary Information

Supplementary Information
