# Supplementary material for: Rational thermostabilisation of four-helix bundle dimeric de novo proteins
Source: Sci Rep. 2021 Apr 6;11:7526. doi: 10.1038/s41598-021-86952-2 (PMC8024369; doi:10.1038/s41598-021-86952-2)
Supplement: Supplementary file 1 — Supplementary Information [file 41598_2021_86952_MOESM1_ESM.pdf]

Supplementary Information

Rational Thermostabilisation of Four-Helix Bundle Dimeric *De Novo* Proteins

Shin Irumagawa[1,2,3], Kaito Kobayashi[4], Yutaka Saito[4,5,6], Takeshi Miyata[7], Mitsuo Umetsu[8], Tomoshi Kameda[4], and Ryoichi Arai[1,2,3]*

[1]Department of Science and Technology, Graduate School of Medicine, Science and Technology, Shinshu University, Ueda 386-8567, Japan.
[2]Department of Biomolecular Innovation, Institute for Biomedical Sciences, Interdisciplinary Cluster for Cutting Edge Research, Shinshu University, Matsumoto 390-8621, Japan.
[3]Department of Applied Biology, Faculty of Textile Science and Technology, Shinshu University, Ueda 386-8567, Japan.
[4]Artificial Intelligence Research Center, National Institute of Advanced Industrial Science and Technology (AIST), Tokyo 135-0064, Japan.
[5]AIST-Waseda University Computational Bio Big-Data Open Innovation Laboratory (CBBD-OIL), Tokyo 169-8555, Japan.
[6]Graduate School of Frontier Sciences, The University of Tokyo, Kashiwa 277-8561, Japan.
[7]Department of Biochemistry and Biotechnology, Faculty of Agriculture, Kagoshima University, Kagoshima 890-0065, Japan.
[8]Department of Biomolecular Engineering, Graduate School of Engineering, Tohoku University, Sendai 980-8579, Japan.

*Corresponding author: e-mail: rarai@shinshu-u.ac.jp

Contents

Supplementary Tables: Tables S1, S2
Supplementary Figures: Figures S1–S14

Amino acids	Chain	Residues	ASA	ASA ratio
GLY	A	3	70.3	0.51
LYS	A	4	137.2	0.64
LEU	A	5	5.4	0.03
ASN	A	6	71.2	0.42
LYS	A	7	152.3	0.70
LEU	A	8	41.1	0.22
VAL	A	9	12.0	0.08
GLU	A	10	102.8	0.53
HIS	A	11	81.0	0.41
ILE	A	12	0.5	0.00
LYS	A	13	66.6	0.31
GLU	A	14	102.1	0.51
LEU	A	15	34.8	0.19
LEU	A	16	5.9	0.03
GLN	A	17	110.0	0.55
GLN	A	18	101.3	0.50
LEU	A	19	4.5	0.02
ASN	A	20	75.1	0.43
LYS	A	21	111.0	0.52
**ASN**	**A**	**22**	**41.5**	**0.24**
TRP	A	23	10.7	0.04
HIS	A	24	137.2	0.69
ARG	A	25	137.2	0.58
HIS	A	26	5.7	0.03
GLN	A	27	55.3	0.28
GLY	A	28	28.6	0.32
ASN	A	29	44.5	0.26
LEU	A	30	4.8	0.03
HIS	A	31	94.1	0.46
ASP	A	32	88.0	0.53
MSE	A	33	9.8	0.05
ASN	A	34	28.1	0.17
GLN	A	35	110.8	0.58
GLN	A	36	69.4	0.36
MSE	A	37	9.2	0.04
GLU	A	38	71.3	0.39
GLN	A	39	109.6	0.58
LEU	A	40	1.8	0.01
PHE	A	41	17.8	0.08
GLN	A	42	82.5	0.43
GLU	A	43	66.1	0.35
PHE	A	44	0.0	0.00
GLN	A	45	53.0	0.26
HIS	A	46	123.7	0.62
PHE	A	47	39.6	0.19
MSE	A	48	38.1	0.19
GLN	A	49	122.6	0.62
GLY	A	50	56.9	0.64
ASN	A	51	25.6	0.16
GLN	A	52	180.4	0.90

Amino acids	Chain	Residues	ASA	ASA ratio
ASP	A	53	71.8	0.49
ASP	A	54	98.9	0.60
GLY	A	55	40.1	0.45
LYS	A	56	115.2	0.55
LEU	A	57	32.0	0.18
GLN	A	58	88.4	0.46
ASN	A	59	108.1	0.63
MSE	A	60	21.1	0.11
ILE	A	61	15.7	0.09
HIS	A	62	91.8	0.48
GLU	A	63	59.7	0.32
MSE	A	64	1.0	0.00
GLN	A	65	80.1	0.40
GLN	A	66	96.0	0.49
PHE	A	67	1.2	0.01
MSE	A	68	4.6	0.02
ASN	A	69	73.8	0.43
GLN	A	70	86.0	0.44
VAL	A	71	4.0	0.03
ASP	A	72	36.3	0.22
ASN	A	73	106.2	0.62
**HIS**	**A**	**74**	**19.9**	**0.11**
LEU	A	75	7.7	0.04
GLN	A	76	84.2	0.45
SER	A	77	50.4	0.42
GLU	A	78	0.0	0.00
**SER**	**A**	**79**	**10.2**	**0.08**
ASP	A	80	89.6	0.53
THR	A	81	22.4	0.15
VAL	A	82	0.1	0.00
HIS	A	83	89.3	0.45
HIS	A	84	103.1	0.53
PHE	A	85	24.6	0.11
**HIS**	**A**	**86**	**12.9**	**0.07**
ASN	A	87	98.2	0.59
LYS	A	88	87.1	0.41
LEU	A	89	6.1	0.03
GLN	A	90	78.5	0.40
GLU	A	91	118.2	0.61
LEU	A	92	15.0	0.08
MSE	A	93	40.7	0.20
ASN	A	94	85.8	0.52
ASN	A	95	72.0	0.42
PHE	A	96	2.6	0.01
HIS	A	97	83.7	0.43
HIS	A	98	126.1	0.63
LEU	A	99	40.4	0.22
VAL	A	100	19.5	0.12
HIS	A	101	150.9	0.62

Table S1. Accessible surface areas (ASA) of amino acid residues in the WA20 structure. The data (chain A) of the crystal structure of WA20 (PDB ID: 3VJF)[16] were calculated by the program AREAIMOL[22,23] in the CCP4 suite[24]. Red letters represent hydrophilic residues with small ASA ratio (ASA ratio ≤ 0.11). (ASA ratio: ratio of ASA to calculated GXG (Gly-Xaa-Gly) value.). The target residues for mutations selected in this study are shown in bold letters.

Amino acids	Chain	Residues	ASA	ASA ratio
LEU	B	5	166.3	0.72
ASN	B	6	8.0	0.04
LYS	B	7	98.7	0.48
LEU	B	8	63.8	0.34
VAL	B	9	3.5	0.02
GLU	B	10	126.5	0.65
HIS	B	11	92.2	0.47
ILE	B	12	2.1	0.01
LYS	B	13	65.6	0.30
GLU	B	14	100.8	0.51
LEU	B	15	44.2	0.24
LEU	B	16	15.5	0.08
GLN	B	17	109.8	0.55
GLN	B	18	121.3	0.60
LEU	B	19	8.5	0.05
ASN	B	20	56.1	0.34
LYS	B	21	153.4	0.70
**ASN**	**B**	**22**	**61.0**	**0.35**
TRP	B	23	2.7	0.01
HIS	B	24	110.8	0.54
ARG	B	25	172.9	0.69
HIS	B	26	3.2	0.02
GLN	B	27	64.4	0.34
GLY	B	28	30.2	0.33
ASN	B	29	51.1	0.30
LEU	B	30	5.6	0.03
HIS	B	31	85.9	0.45
ASP	B	32	66.5	0.40
MSE	B	33	7.0	0.04
ASN	B	34	27.4	0.17
GLN	B	35	87.3	0.46
GLN	B	36	42.4	0.22
MSE	B	37	8.8	0.04
GLU	B	38	78.4	0.44
GLN	B	39	109.3	0.56
LEU	B	40	0.2	0.00
PHE	B	41	23.1	0.11
GLN	B	42	87.5	0.46
GLU	B	43	38.6	0.21
PHE	B	44	0.1	0.00
GLN	B	45	89.5	0.46
HIS	B	46	145.7	0.74
PHE	B	47	105.2	0.47
MSE	B	48	112.3	0.47
LYS	B	56	186.6	0.71
LEU	B	57	52.5	0.28
GLN	B	58	104.5	0.53
ASN	B	59	101.9	0.60
MSE	B	60	54.7	0.27
ILE	B	61	13.4	0.07
HIS	B	62	100.9	0.52
GLU	B	63	75.9	0.40
MSE	B	64	4.7	0.02
GLN	B	65	46.1	0.23
GLN	B	66	95.5	0.49
PHE	B	67	1.6	0.01
MSE	B	68	5.6	0.03
ASN	B	69	61.5	0.36
GLN	B	70	96.5	0.48
VAL	B	71	1.6	0.01
ASP	B	72	31.4	0.19
ASN	B	73	103.5	0.61
**HIS**	**B**	**74**	**9.7**	**0.05**
LEU	B	75	7.5	0.04
GLN	B	76	82.4	0.45
SER	B	77	46.7	0.35
GLU	B	78	0.0	0.00
**SER**	**B**	**79**	**2.3**	**0.02**
ASP	B	80	84.9	0.51
THR	B	81	17.4	0.12
VAL	B	82	0.1	0.00
HIS	B	83	69.9	0.35
HIS	B	84	93.6	0.48
PHE	B	85	13.5	0.06
**HIS**	**B**	**86**	**14.1**	**0.07**
ASN	B	87	90.2	0.54
LYS	B	88	76.4	0.36
LEU	B	89	6.9	0.04
GLN	B	90	82.7	0.42
GLU	B	91	103.7	0.52
LEU	B	92	14.9	0.08
MSE	B	93	45.8	0.22
ASN	B	94	83.4	0.51
ASN	B	95	81.6	0.47
PHE	B	96	5.6	0.03
HIS	B	97	90.8	0.47
HIS	B	98	122.3	0.61
LEU	B	99	49.0	0.26
VAL	B	100	36.2	0.22
HIS	B	101	160.4	0.66

Table S1 (continued). Accessible surface areas (ASA) of amino acid residues in the WA20 structure. The data (chain B) of the crystal structure of WA20 (PDB ID: 3VJF)[16] were calculated by the program AREAIMOL[22,23] in the CCP4 suite[24]. Red letters represent hydrophilic residues with small ASA ratio (ASA ratio ≤ 0.11). (ASA ratio: ratio of ASA to calculated GXG (Gly-Xaa-Gly) value.) The target residues for mutations selected in this study are shown in bold letters.

Primer name	Sequence (5’→3’)
T7 promoter primer	TAATACGACTCACTATAGGG
T7 terminator primer	GCTAGTTATTGCTCAGCGG
WA20_N22A_Fw	TGCAACAGTTGAACAAAGCCTGGCACAGGC
WA20_N22E_Fw	TGCAACAGTTGAACAAAGAATGGCACAGGC
WA20_N22K_Rv	GCCTGTGCCACTTTTTGTTCAACTGTTGCAGCAATTC
WA20_N22L_Rv	GCCTGTGCCAGAGTTTGTTCAACTGTTGCAGCAATTC
WA20_H86K_Fw	GACACCGTGCATCATTTCAAGAACAAATTGCAGGAGC
WA20_H86S_Fw	GACACCGTGCATCATTTCAGCAACAAATTGCAGGAGC
WA20_H74V_S79F_ Rv	GAAATGATGCACGGTGTCGAACTCGCTCTGCAGAACGTTGTCGACTTGGTTCATGAACTG

Table S2. Oligonucleotide primer sequences used in this study.
Red letters correspond to the codons for amino acid substitutions.

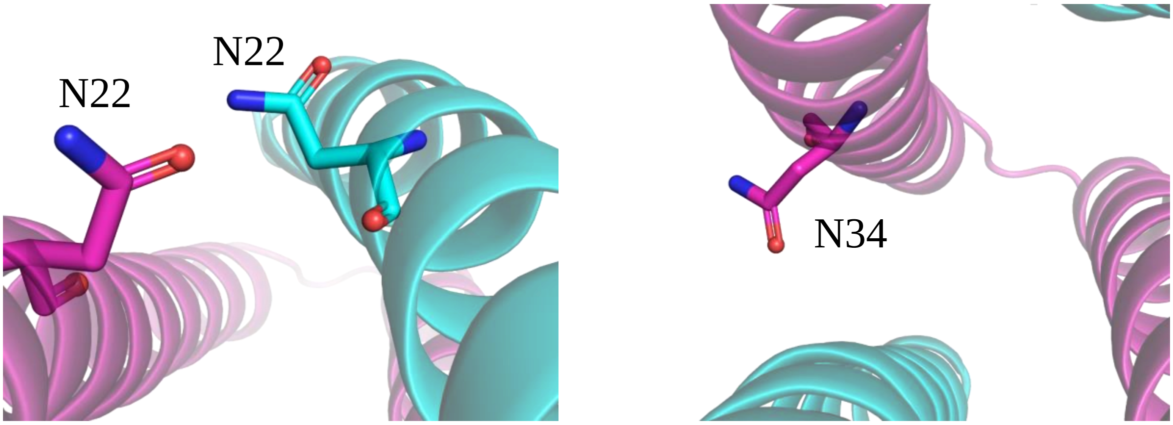

Figure S1. Potential target residues on the interface of α-helices in the WA20 structure. Two target residues (N22 and N34) on the interface of α-helices were chosen to potentially enhance helix-helix interactions. The target residues are shown as sticks. Chains A and B of the crystal structure of WA20 (PDB: 3VJF)[16] are shown in magenta and cyan, respectively. The images were created using open-source PyMOL, version 2.4 (https://github.com/schrodinger/pymol-open-source).

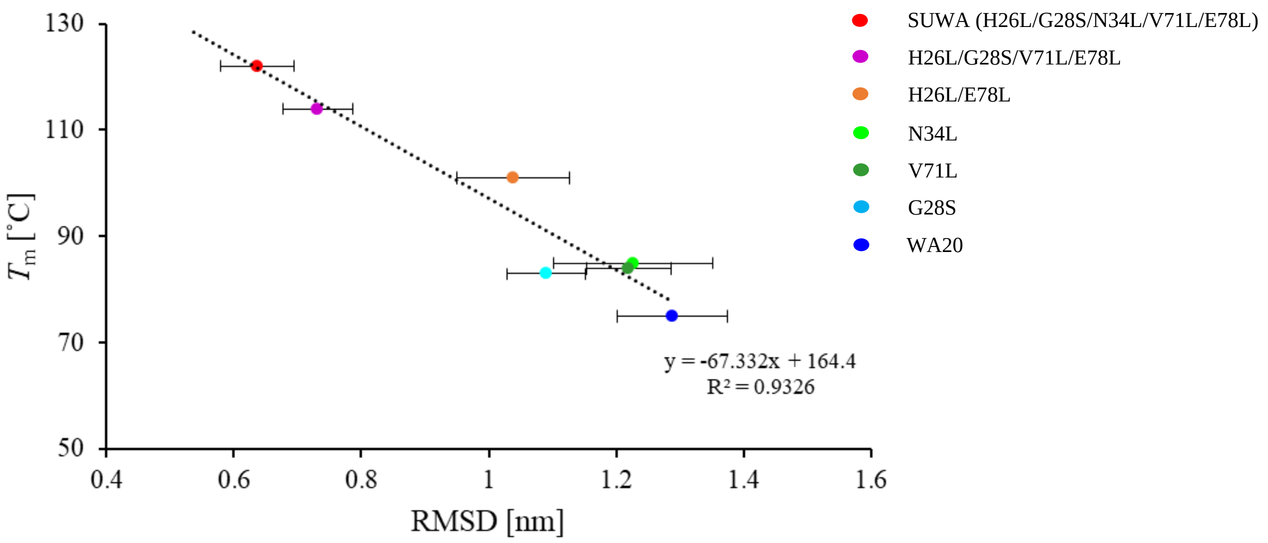

Figure S2. Correlation of experimental T_m and RMSD calculated by high-temperature MD simulations for WA20 mutants.

MD simulations under high temperature condition (600 K, 10 ns) were performed for WA20 and the thermostabilised mutants reported in a previous study[20]. Root mean square deviations (RMSD) between initial structures and structures after high-temperature MD simulations were calculated to evaluate the degrees of protein unfolding. MD simulations were performed ten times for each protein. There is a strong negative correlation between RMSD and experimentally determined T_m of WA20 and the mutants ($r = -0.966$, $p = 5.461 \times 10^{-5}$).

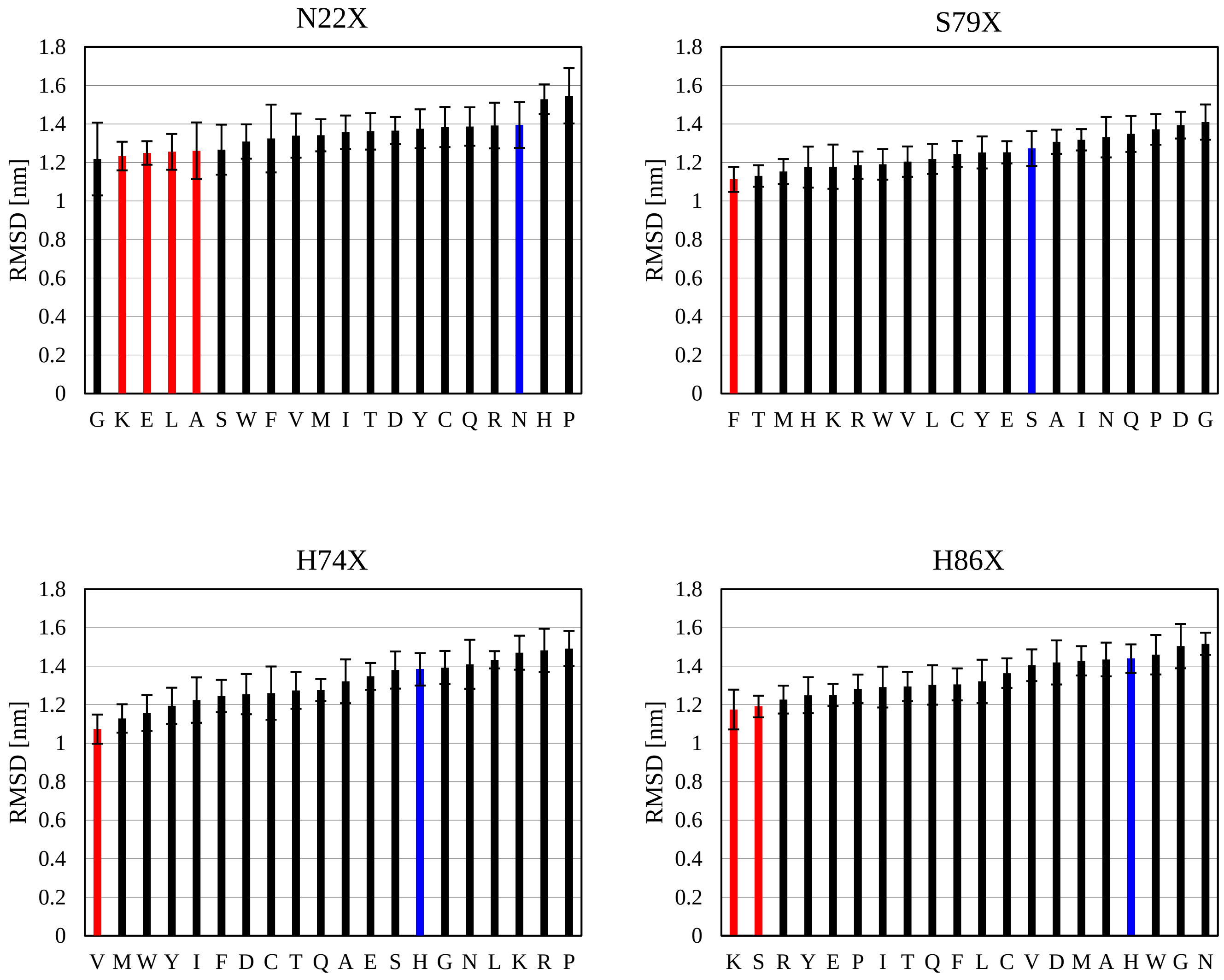

Figure S3. RMSD of the WA20 mutant structures calculated by high-temperature MD simulations. MD simulations under high temperature condition (600 K, 10 ns) were performed for WA20 and all of the possible single mutants of WA20 generated by *in silico* saturation mutagenesis at the four target residue sites. Root mean square deviations (RMSDs) between the initial structures and the structures after high-temperature MD simulations were calculated to evaluate the degrees of protein unfolding. MD simulations were performed ten times for each mutant. Blue bars represent the original WA20. Red bars of small RSMD values represent the mutants selected for experimental evaluations in this study.

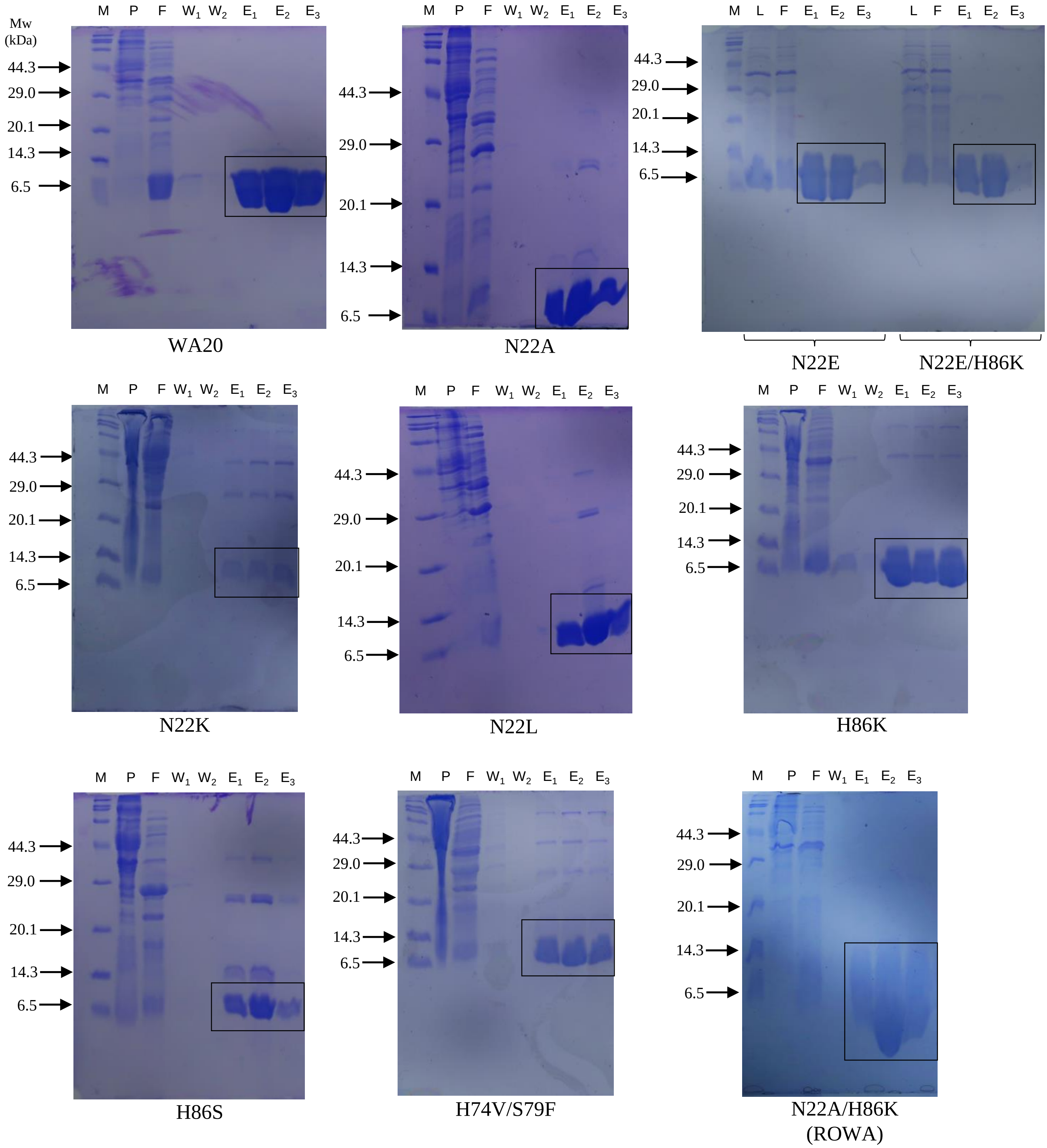

Figure S4. SDS-PAGE analysis of WA20 and the WA20 mutants.
Sodium dodecyl sulfate polyacrylamide gel electrophoresis (SDS-PAGE) analysis of the WA20 mutants were performed for the eluted fractions by immobilised metal ion affinity chromatography (IMAC) with TALON metal affinity resin (Takara Bio, Otsu, Japan). The protein bands surrounded in the black square show purified WA20 and the mutants (molecular mass of a monomer of WA20 and its mutants: ~12.5 kDa). Protein molecular weight markers (broad) (Takara Bio) are shown in left. Proteins were stained with Coomassie brilliant blue.
Abbreviations: M, molecular weight markers; P, pellet fraction; L, lysate; F, flow-through fraction; W, wash fractions; E, eluted fractions.

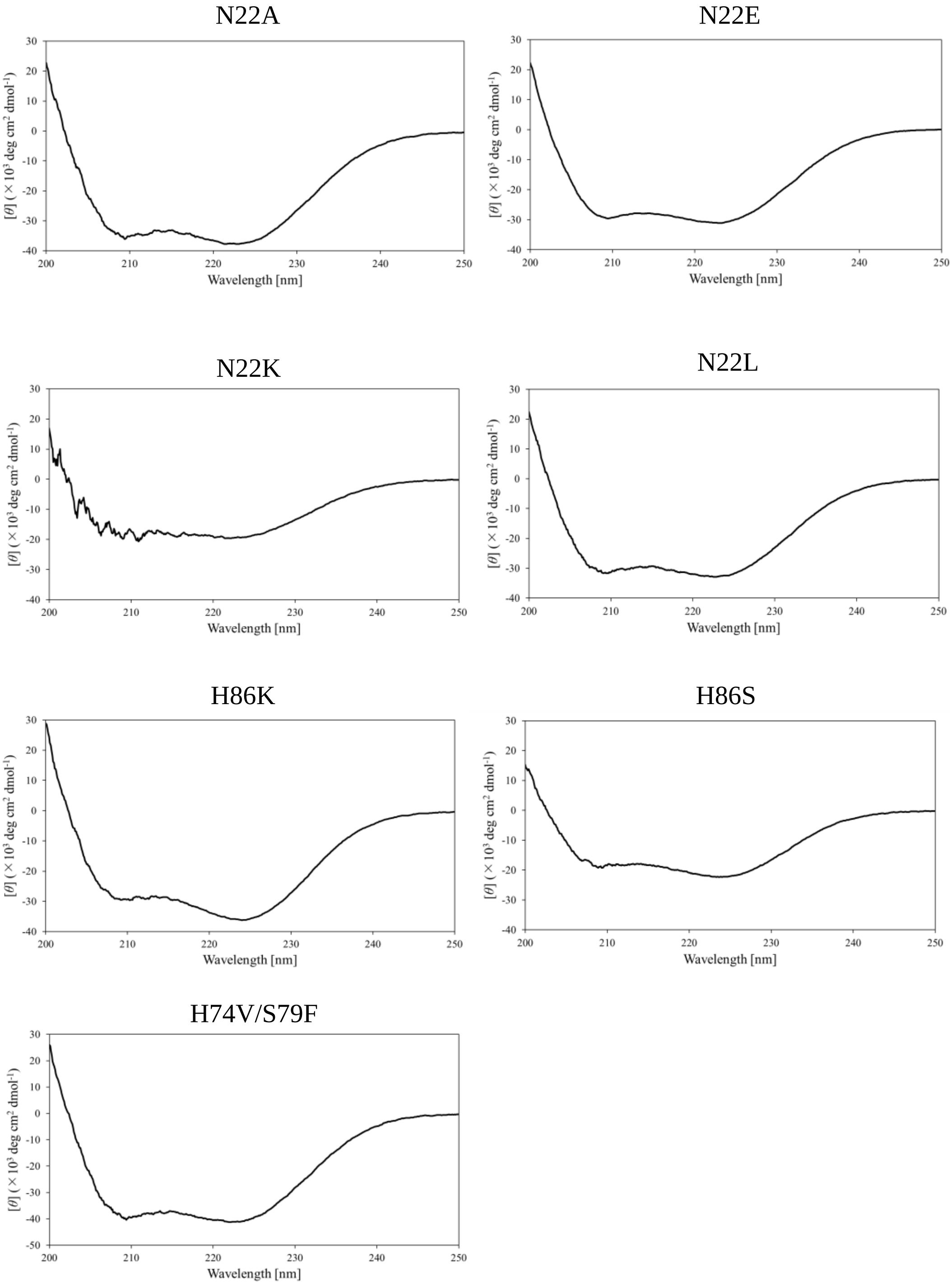

Figure S5. Circular dichroism (CD) spectra of the WA20 mutants.
CD spectra were measured at 25 °C.

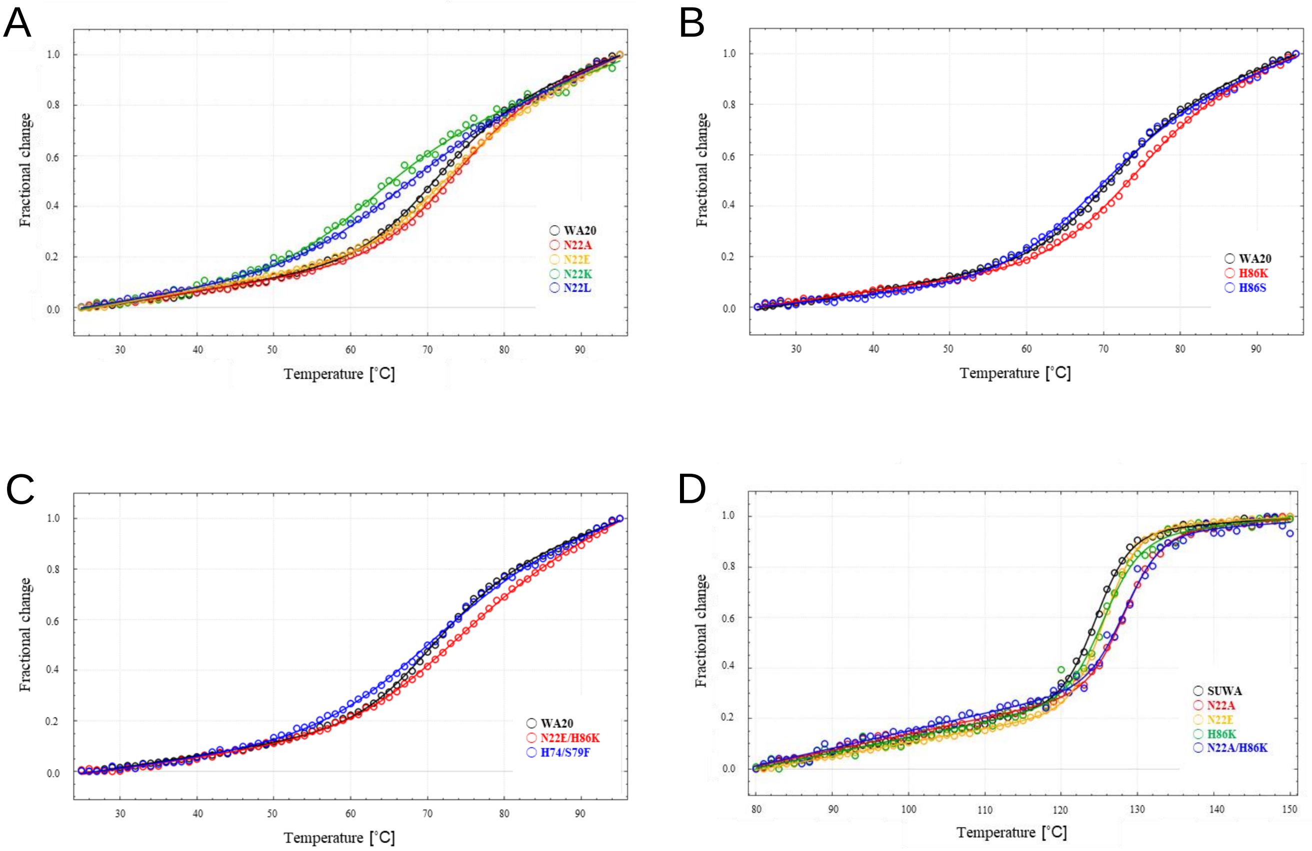

Figure S6. Thermal denaturation curves of (A, B, C) WA20 mutants and (D) SUWA mutants. The *Y*-axis represents the normalised value of $[\theta]_{222\ nm}$. The experimental data were fitted to a two-state model with ΔC_p fixed to zero using CDpal[33]. The T_m values of the WA20 mutants and the SUWA mutants are shown in Table 1 and Table 2, respectively. The graphs were created using CDpal[33], version 2.18 (https://github.com/PINT-NMR/CDpal/).

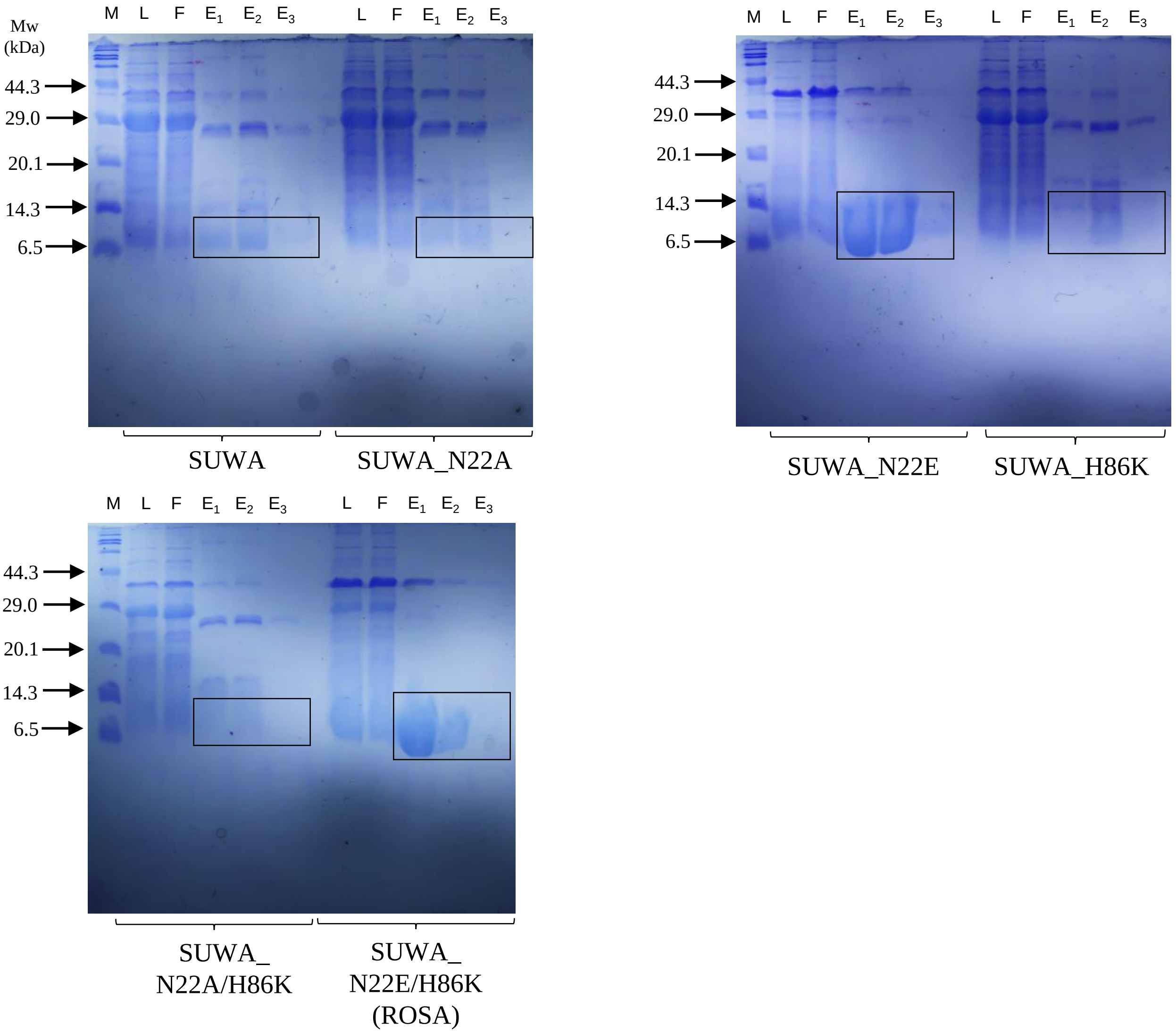

Figure S7. SDS-PAGE analysis of SUWA and the SUWA mutants.
SDS-PAGE analysis of SUWA and the SUWA mutants were performed for the eluted fractions by IMAC with TALON metal affinity resin (Takara Bio). The protein bands surrounded in the black square show purified SUWA and the mutants (molecular mass of a monomer of SUWA and its mutants: ~12.6 kDa). Protein molecular weight markers (broad) (Takara Bio) are shown in left. Proteins were stained with Coomassie brilliant blue.
Abbreviations: M, molecular weight markers; L, lysate; F, flow-through fraction; E, eluted fractions.

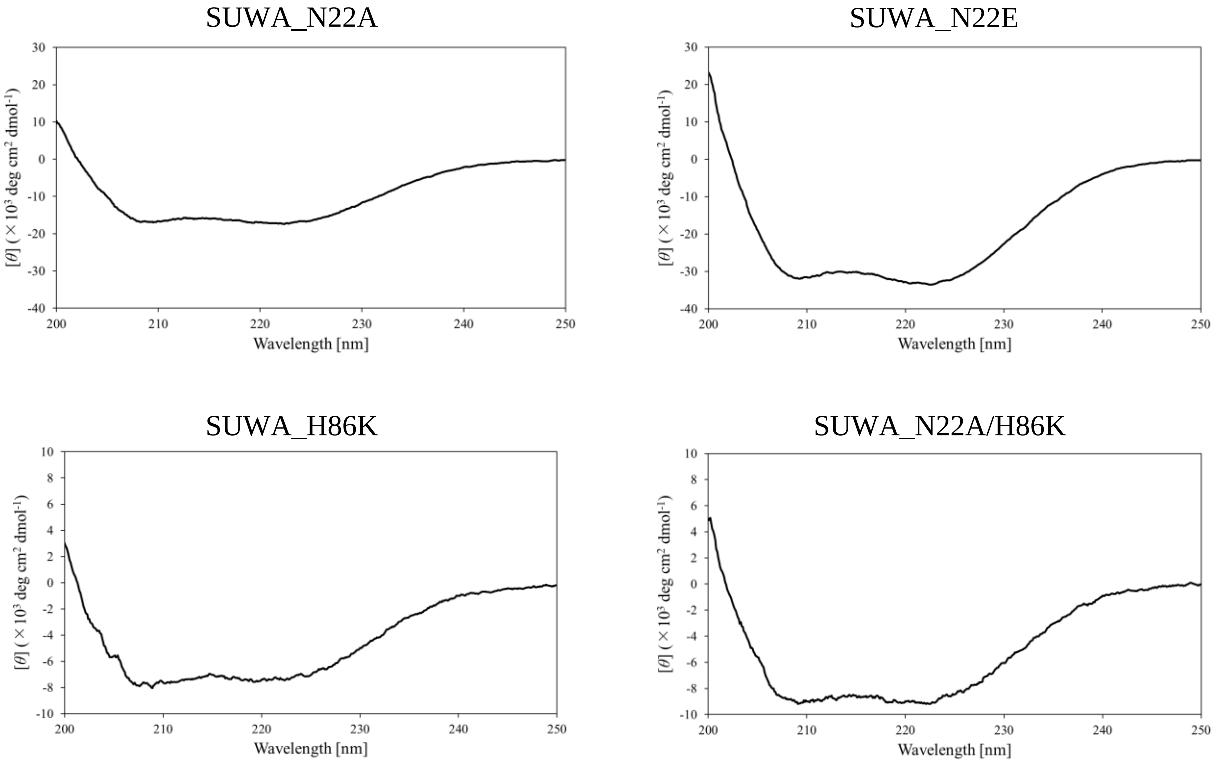

Figure S8. Circular dichroism (CD) spectra of the SUWA mutants
CD spectra were measured at 30 °C.

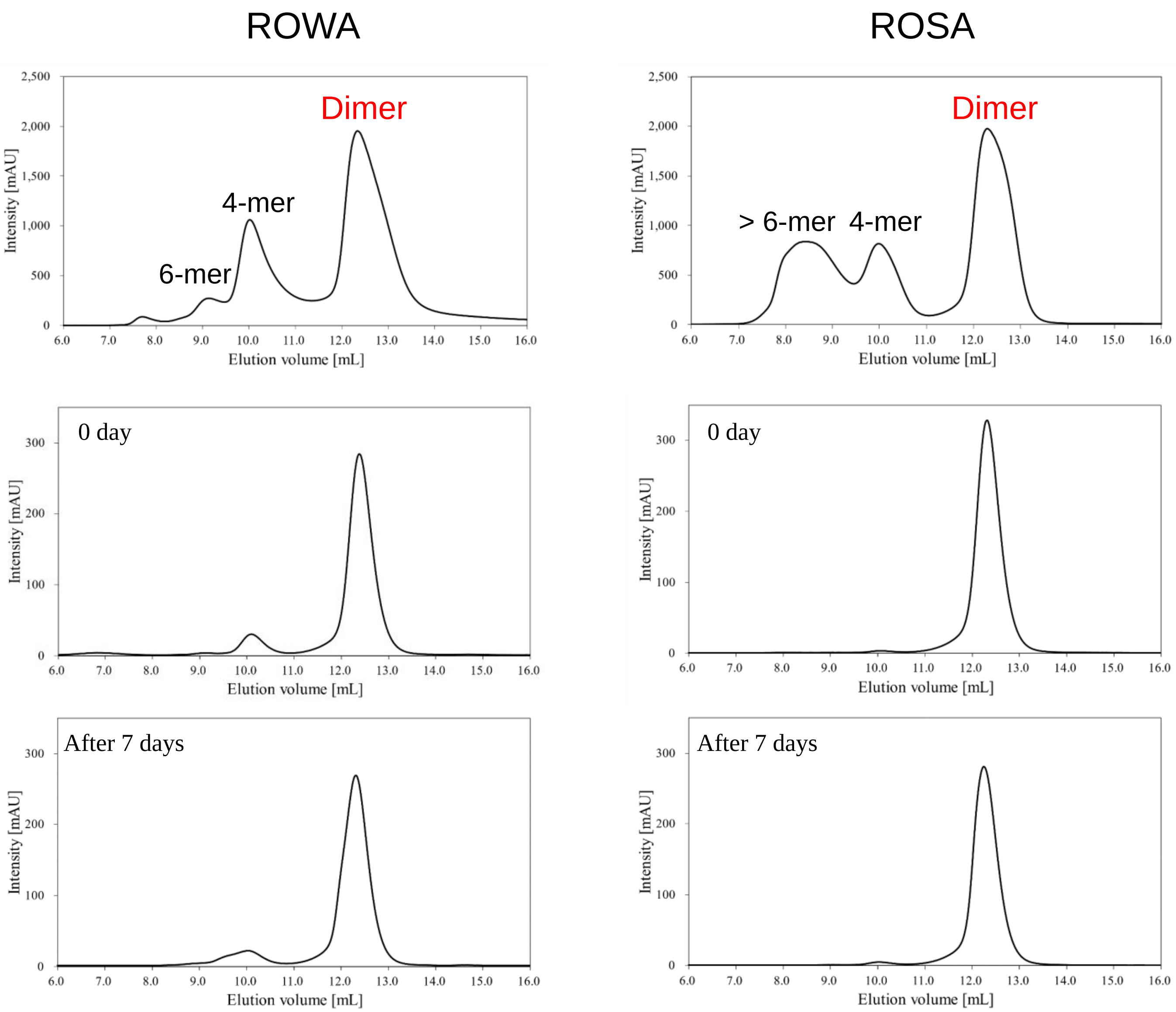

Figure S9. Size exclusion chromatography (SEC) analyses of ROWA and ROSA dimers. SEC profiles of IMAC-purified samples of ROWA (left) and ROSA (right) with a Superdex 75 increase 10/300 GL column (Cytiva) are shown in top panels. The isolated fractions of the dimer peaks of ROWA and ROSA were reanalysed immediately after the SEC purification (0 day, middle panels) and after keeping them at 20 °C for 7 days (bottom panels). These results suggest that the dimers did not change to the other oligomers, i.e., the oligomeric states do not exchange one another on a timescale of a week. The *Y*-axis represents the intensity of UV absorbance ($A_{280\ nm}$).

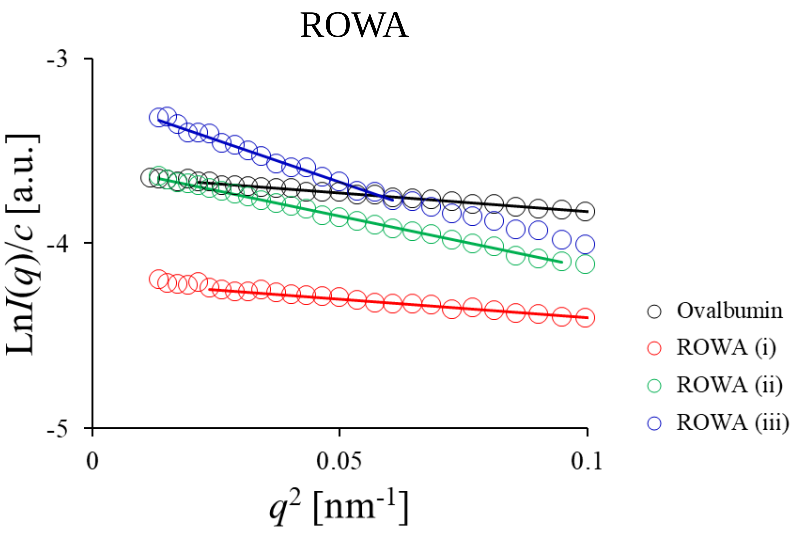

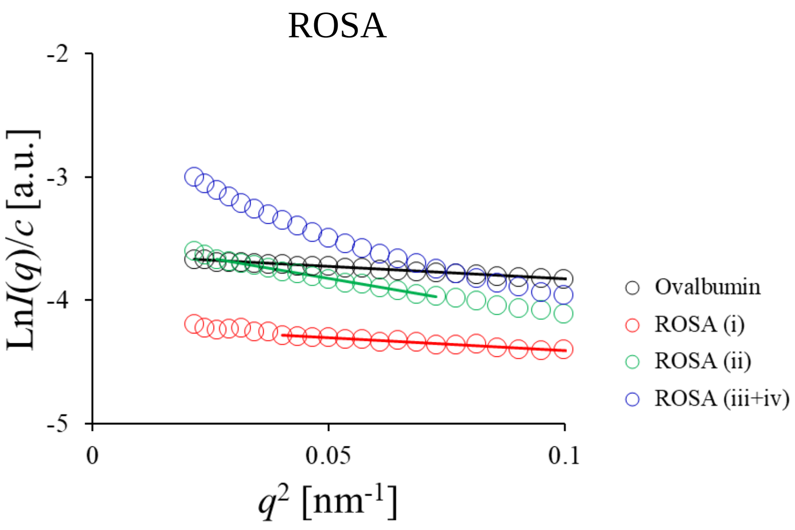

Figure S10. Guinier plots of the SAXS data of the ROWA and ROSA samples.
Forward scattering intensity, $I(q \rightarrow 0)$, and radius of gyration, R_g, were estimated by Guinier approximation[39] using AUTORG in ATSAS[38] with SAngler[36]. However, the Guinier approximation was not applied to the data of the ROSA (iii+iv) sample because the linearity of the Guinier plot of ROSA (iii+iv) was not good enough for a linear approximation probably due to polydispersity or aggregation.

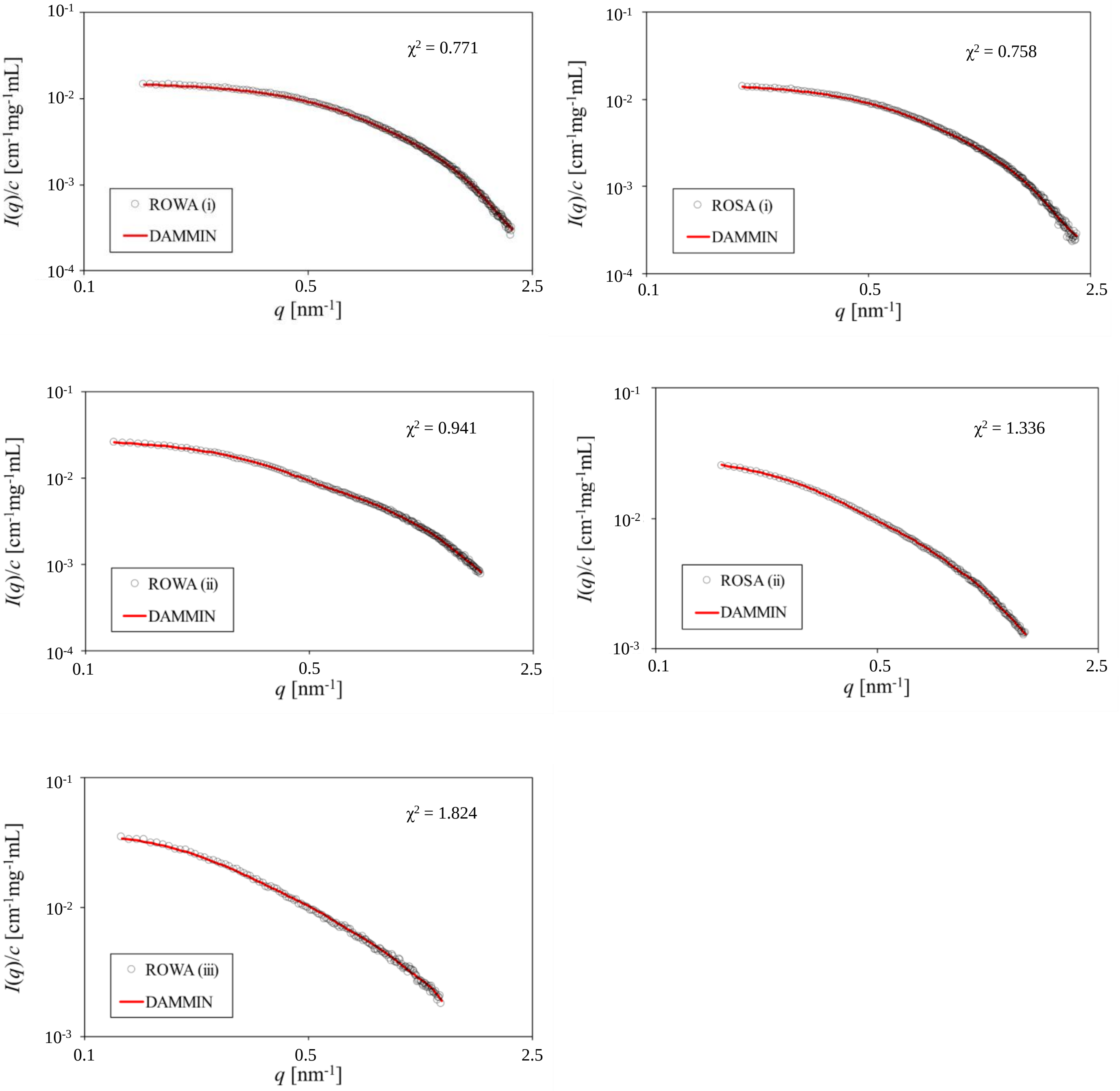

Figure S11. Plots of the scattering curves calculated from the DAMMIN models fitting to the experimental SAXS data.

The concentration-normalised SAXS intensity, $I(q)/c$, of the ROWA and ROSA samples (black open circle) and that optimised by the DAMMIN procedure (red line). The χ^2 value represents the degree of fitting between the experimental data and the data calculated from the DAMMIN model.

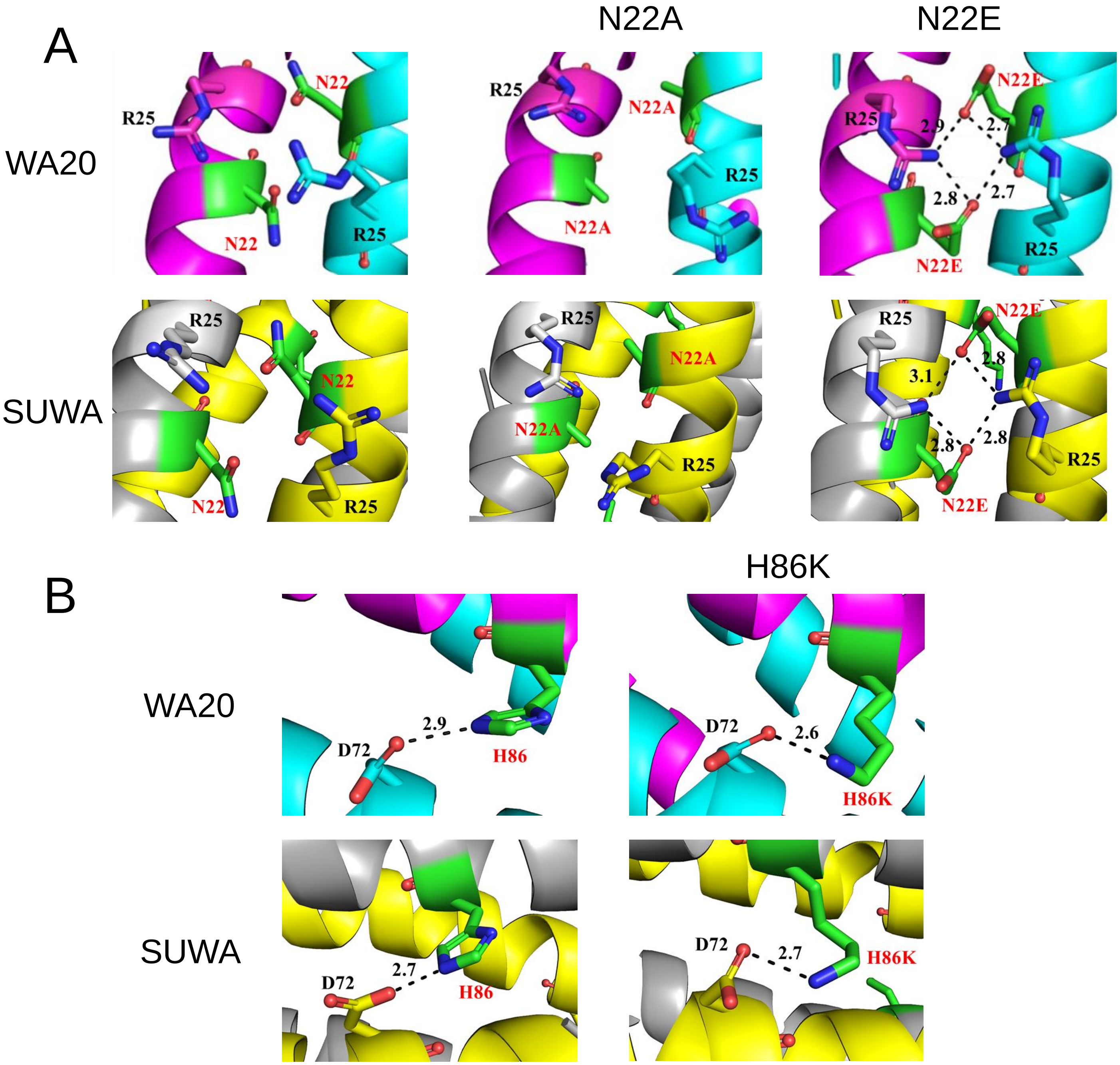

Figure S12. Model structures of the thermostabilised mutants of WA20 and SUWA.
(A) Model structures of the N22A and N22E mutants of WA20 and SUWA. The left panels show the structures of WA20 and SUWA and the middle and right panels show the model structures of the mutants. Chains A and B of WA20 are shown in magenta and cyan, respectively. Chains A and B of SUWA are shown in gray and yellow, respectively. The target and mutated residues are shown as green sticks. The interatomic distances between oxygen of E22 and nitrogen of R25 (Å) are shown with dashed lines. (B) Model structures of the H86K mutants of WA20 and SUWA. Chains A and B of WA20 are shown in magenta and cyan, respectively. Chains A and B of SUWA are shown in gray and yellow, respectively. The target and mutated residues are shown as green sticks. The interatomic distances between oxygen of D72 and nitrogen of H86 or K86 (Å) are shown with dashed lines. The model structures were constructed from the crystal structures of WA20 (PDB ID: 3VJF) or SUWA (PDB ID: 6KOS) and optimised by MD simulation at 300 K for 1 ns.
The images were created using open-source PyMOL, version 2.4 (https://github.com/schrodinger/pymol-open-source).

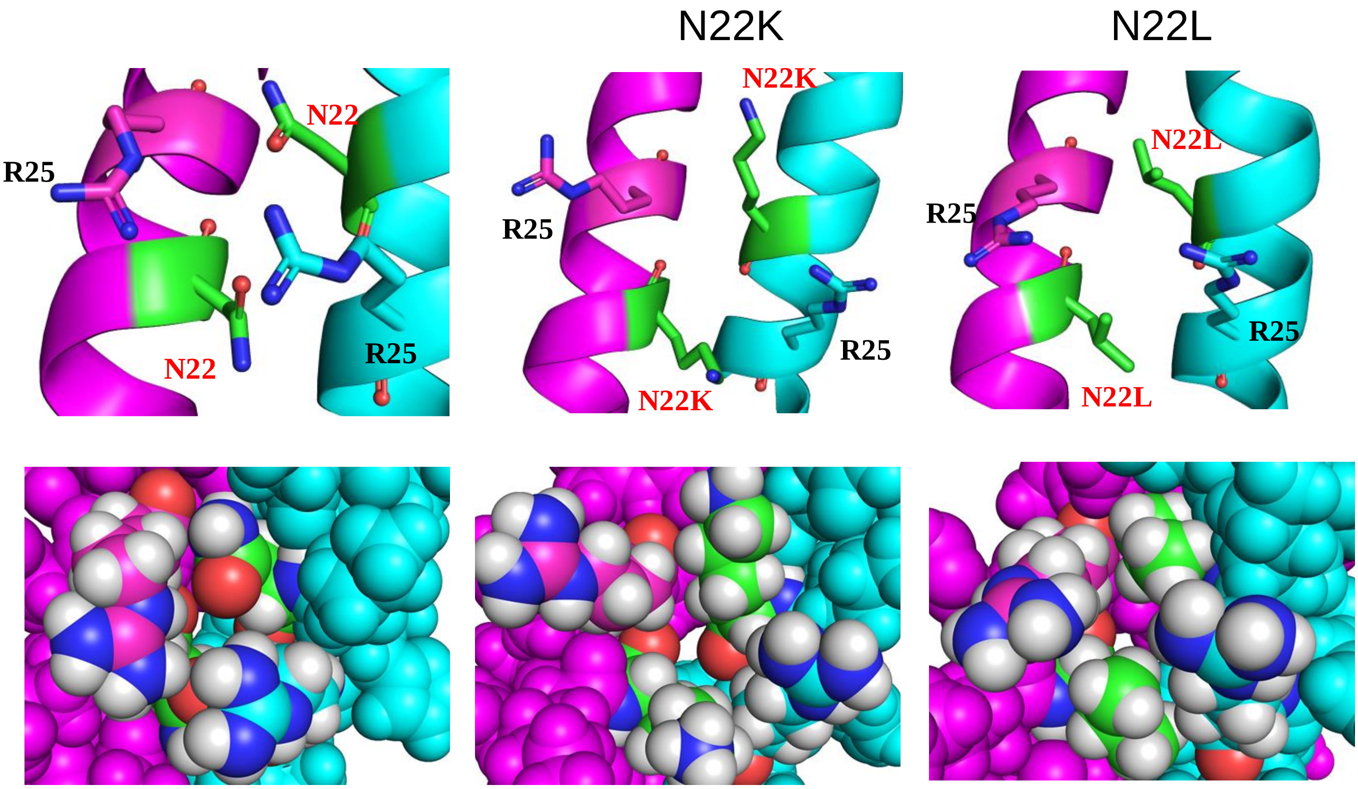

Figure S13. Model structures of the destabilised mutants of WA20.
The left panels show the structure of WA20. The middle and right panels show the model structures of the N22K and N22Lmutants, respectively. Chains A and B of WA20 are shown in magenta and cyan, respectively. The target and mutated residues are shown as green sticks in the upper panels. In the lower panels, all atoms including hydrogens are shown as spheres, and colors are the same as the upper panels. The model structures were constructed from the crystal structure of WA20 (PDB ID: 3VJF) and optimised by MD simulation at 300 K for 1 ns.
The images were created using open-source PyMOL, version 2.4 (https://github.com/schrodinger/pymol-open-source).

WA20

MYGKLNKLVE HIKELLQQLN KNWHRHQGNL HDMNQQMEQL
FQEFQHFMQG NQDDGKLQNM IHEMQQFMNQ VDNHLQSESD
TVHHFHNKLQ ELMNNFHHLV HR

(Molecular mass of a monomer: 12547)

ROWA (N22A/H86K)

MYGKLNKLVE HIKELLQQLN K**A**WHRHQGNL HDMNQQMEQL
FQEFQHFMQG NQDDGKLQNM IHEMQQFMNQ VDNHLQSESD
TVHHF**K**NKLQ ELMNNFHHLV HR

(Molecular mass of a monomer: 12495)

SUWA (H26L/G28S/N34L/V71L/E78L)

MYGKLNKLVE HIKELLQQLN KNWHR**L**Q**S**NL HDM**L**QQMEQL
FQEFQHFMQG NQDDGKLQNM IHEMQQFMNQ **L**DNHLQS**L**SD
TVHHFHNKLQ ELMNNFHHLV HR

(Molecular mass of a monomer: 12550)

ROSA (N22E/H26L/G28S/N34L/V71L/E78L/H86K)

MYGKLNKLVE HIKELLQQLN K**E**WHR**L**Q**S**NL HDM**L**QQMEQL
FQEFQHFMQG NQDDGKLQNM IHEMQQFMNQ **L**DNHLQS**L**SD
TVHHF**K**NKLQ ELMNNFHHLV HR

(Molecular mass of a monomer: 12556)

Figure S14. Amino acid sequences of WA20, ROWA, SUWA, and ROSA.
Red letters represent the stabilising mutations found in this study. Blue letters represent the stabilising mutations reported in a previous study[20].
